# Gender-Affirming Surgery in Low- and Middle-Income Countries: A Systematic Review

**DOI:** 10.3390/jcm13123580

**Published:** 2024-06-19

**Authors:** Viraj Shah, Bashar Hassan, Rena Hassan, Malory Alexis, Myan Bhoopalam, Lorreen Agandi, Fan Liang

**Affiliations:** 1 Faculty of Medicine, Imperial College London, London SW10 9NH, UK; thevirajshah@outlook.com; 2 Johns Hopkins Medicine, Baltimore, MD 21287, USA; bhassan2@jh.edu (B.H.); mbhoopa1@jhmi.edu (M.B.); 3 Department of Plastic and Reconstructive Surgery, Center for Transgender and Gender Expansive Health, Johns Hopkins Medicine, 600 North Wolfe Street, Baltimore, MD 21287, USA; 4 Faculty of Medicine, Saint Georges University of Beirut, Beirut 2807, Lebanon; reina.awad.hasan@gmail.com; 5 Florida State University College of Medicine, Tallahassee, FL 32301, USA; malory1.alexis@gmail.com; 6 Touro College of Osteopathic Medicine, New York, NY 10027, USA; lagandi@student.touro.edu

**Keywords:** gender-affirming surgery, transgender, non-binary, global surgery, LMICs

## Abstract

**Objectives:** Fewer than one-fifth of all studies on gender-affirming care originate from low- and middle-income countries (LMICs). This is the first systematic review to examine surgical demographics and outcomes following gender-affirming surgery (GAS) in LMICs. **Methods:** Following the Preferred Reporting Items for Systematic Reviews and Meta-Analyses guidelines, five databases were systematically searched for original studies and case series on GAS within LMIC settings. Excluded reports included animal studies, non-English language studies, secondary studies including reviews, individual case reports and conference abstracts. **Results:** This review includes 34 studies involving n = 5064 TGNB individuals. Most studies (22, 64.7%) were from upper-middle-income countries, followed by lower-middle-income countries (12, 35.3%). A total of 31 studies (91.2%) reported on post-operative outcomes. Of n = 5013 patients who underwent GAS, 71.5% (n = 3584) underwent masculinizing and 29.5% (n = 1480) underwent feminizing procedures. The predominant procedures were metoidioplasty (n = 2270/3584, 63.3%) and vaginoplasty (n = 1103/1480, 74.5%). Mean follow-up was 47.7 months. In patients who underwent metoidioplasty, 6.8% (n = 155) of patients experienced a complication and 6.3% (n = 144) underwent revision surgery. In patients who underwent vaginoplasty, 11.5% (n = 127) of patients experienced a complication and 8.5% (n = 94) underwent revision surgery. Of the studies (25/34, 73.5%) that reported on quality of life and post-operative satisfaction, the majority showed marked improvements in psychosocial and functional outcomes. Notably, no post-surgical regret was reported among the surveyed patients. **Conclusions:** Existing literature on GAS in LMICs remains scarce and is concentrated in select institutions that drive specific procedures. Our review highlights the low reported volumes of GAS, variability in surgical outcomes and quality of life.

## 1. Introduction

Gender-affirming surgery (GAS), which allows the alignment of gender identity with physical appearance [1], is recognized as a therapeutic intervention and a medical necessity for many transgender and non-binary (TGNB) individuals with gender dysphoria [2,3,4,5,6]. Over the past decade, the incidence of individuals receiving GAS in high-income countries (HICs) has increased, likely due to expansion in private and government insurance, changes in societal attitudes, and legislative advancements [7]. However, data and trends are harder to ascertain in non-HIC countries, where the true prevalence of TGNB identities is frequently underreported due to stigma, and where published literature is less prolific [7,8]. In a scoping review of peer-reviewed quantitative articles discussing TGNB health from 2008 to 2014, under one-fifth (22/116) were from low-income and middle-income countries (LMICs), and only nine studies addressed gender-affirming surgical care [8].

While there are more than 300 million surgical procedures performed around the world annually, an estimated 5 billion people are unable to access safe surgical treatment. Up to 94% of those with limited access live in LMICs [9]. The International Surgical Outcomes Study evaluated incidence and risk factors for complications and death after inpatient elective surgery at 474 hospitals in 27 countries of varied economic status [10]. Data demonstrated that barriers to providing safe surgical treatment in LMICs included low hospital procedural volumes, few hospital beds, and a scarce number of operating theatres, all compounded by the geographical remoteness of many surgical hospitals and an absence of adequately trained staff [10]. In addition, LMICs have fewer reliable systems to monitor the volume of activity and surgical outcomes. For GAS in particular, impediments to care are compounded by the dearth of adequately trained surgeons, cultural misperceptions, stigma, the need for ongoing post-operative care, and high levels of surgical complexity, particularly with relation to genital surgery [11].

Understanding of the safety, outcomes, and effectiveness of GAS in LMICs is limited, highlighting the need for robust auditing and public reporting of surgical outcomes following GAS in these countries. This review seeks to provide a comprehensive and general overview of current volumes, surgery distribution, and post-operative outcomes of GAS in LMICs. We also aim to identify the challenges to performing GAS in LMICs and suggest future recommendations to improve care in these countries.

## 2. Methods

This systematic review adhered to the Preferred Reporting Items for Systematic Reviews and Meta-Analyses (PRISMA) [12] and Cochrane [13] guidelines to analyze pre-operative demographics and outcomes for GAS in TGNB individuals in LMICs. The search was conducted using EMBASE, MEDLINE, Cochrane, PubMed, and Google Scholar databases, as outlined in Supplementary Material Document S1. We aimed to identify all original articles and case series from inception to December 2023 involving GAS in LMICs. In addition to articles obtained through the database search, some articles were identified by examining the reference lists of those found in the initial search. The only limitations applied to the search were the timeframe and English language.

In our review, countries were classified as LMICs according to the World Bank’s 2023–2024 gross national income (GNI) classifications [14]. The World Bank’s annual classification stratifies countries by GNI per capita, defining low-income countries as those with a per-capita GNI of less than USD 1135, lower-middle-income countries as those with a per-capita GNI between USD 1136 and USD 4465, and upper-middle-income countries as those with a per-capita GNI between USD 4466 and USD 13,845. Therefore, an LMIC is defined as any country meeting these classifications. Supplementary Material Document S2 provides a full list of LMICs that met these criteria and were included in our review.

### 2.1. Inclusion and Exclusion Criteria

All original articles that reported on GAS outcomes within LMICs were considered for review. Inclusion criteria encompassed any original studies or case series (where n > 5) that reported on the performance of GAS in patient populations, with either pre-operative demographic data and/or post-operative outcomes. Exclusion criteria included studies meeting the following conditions: (1) data were inconsistent or of ambiguous quality, hindering data extraction; (2) studies were non-clinical or conducted using animal models; (3) studies lacked explicit reference to GAS; (4) procedures occurred in HICs; (5) studies were editorials, reviews, individual case reports with single patients, preclinical investigations, or meeting abstracts; and (6) duplicate articles. Following the initial review of titles and abstracts, two independent reviewers (RH and LA) conducted a secondary assessment to determine the inclusion and exclusion of articles. A third independent reviewer (MA) resolved any disagreements between the original reviewers. Eligible studies were then retrieved for full-text assessment.

### 2.2. Data Extraction

The following data were extracted: first author, study design, study country, country’s income classification, primary outcome, number of patients, and patient demographics (if applicable), including gender identity, history of smoking, hormone therapy, and mental health therapy. We also captured duration of follow-up, type of procedure received, post-operative outcomes (e.g., complications, success and revision rates, and post-operative satisfaction and/or quality of life when reported). One author performed data extraction (VS) and a third independent reviewer (BH) assessed both full-texts and the data extraction sheet for discrepancies and resolved any disagreements. The studies included in this review are markedly heterogeneous; as a result, quality assessment using formalized scoring tools (e.g., Cochrane’s Risk of Bias Assessment) was not performed [15].

## 3. Results

### 3.1. Study Selection and Assessment

**Figure 1:**
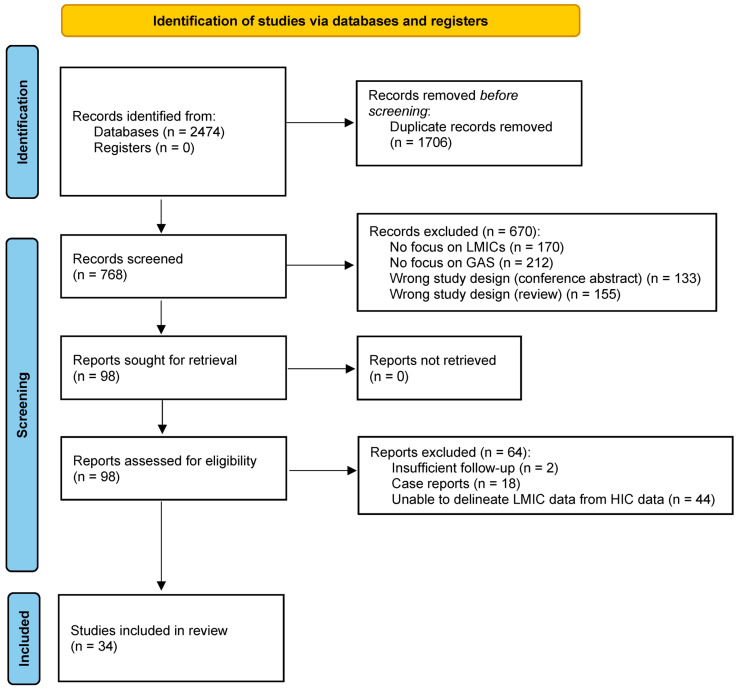
PRISMA Flow Diagram for Inclusion and Exclusion of Studies [12].

The literature search identified 768 articles, following the removal of duplicates from an original 2474 articles. Following abstract screening, 98 articles underwent full-text review and assessment in accordance with the inclusion/exclusion criteria. Following final review, a total of 34 studies [16,17,18,19,20,21,22,23,24,25,26,27,28,29,30,31,32,33,34,35,36,37,38,39,40,41,42,43,44,45,46,47,48,49], encompassing n = 5064 patients, were included in the final analysis. Figure 1 shows the PRISMA flow diagram.

Table 1 provides a concise summary of the included studies, detailing their country of origin, study design, complications, domains assessed for post-operative satisfaction, and follow-up duration.

### 3.2. Temporal Trends and Geographic Disparities in Gender-Affirming Surgery Literature

A total of 23 (67.6%) studies were published during the 5 years preceding this review’s search end-date, while 14 (41.1%) were published during the 2 years prior to this review. Only 2 (5.9%) studies were published before 2010. This reflects a positive temporal trend, with a recent rapid increase in the GAS literature from LMICs.

**Figure 2:**
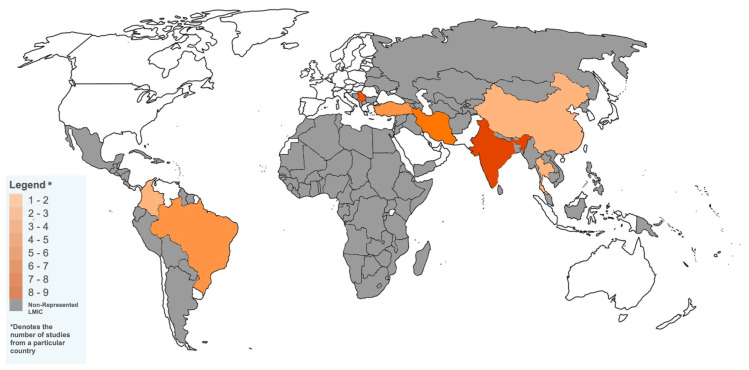
A World Map Illustrating Low and Middle-Income Countries Based on GAS Literature Output.

A total of 12 studies (35.3%) [17,18,19,21,29,31,32,33,34,35,39,46] were from lower-middle-income countries and 22 studies (64.7%) [16,20,22,23,24,25,26,27,28,30,36,37,38,40,41,42,43,44,45,47,48,49] were from upper-middle-income countries. No studies originated from low-income countries. Figure 2 displays a global map illustrating the LMICs included in this review, along with the corresponding number of publications from each country. Five studies involving n = 201 patients originated from Turkey, where GAS is not legalized, and an explicit court order must be sought for provision of services [16,20,41,42,49].

### 3.3. Patient Characteristics and Surgical Outcomes

The mean patient age was 30 years (absolute range: 24.4–41.0). A total of 16 (47.1%) studies included patients’ medical histories [16,17,20,21,22,23,29,31,38,39,40,41,44,46,47,49] and 31 (91.2%) reported on post-operative outcomes [16,18,19,20,21,22,23,24,25,27,28,29,30,31,32,33,34,35,36,37,38,40,41,42,43,44,45,46,47,48,49]. The mean post-operative follow-up was 48 months (absolute range: 6–94 months). Of n = 5064 TGNB patients reported across the review as surgical candidates, n = 5013 (99%) were confirmed as successfully completing GAS. Of these, 71.5% (n = 3584) underwent masculinizing and 29.5% (n = 1480) underwent feminizing procedures.

#### 3.3.1. Masculinizing GAS

A total of n = 18 studies [17,18,21,24,25,26,30,34,36,37,38,39,40,41,42,47,48,49] included patients who underwent masculinizing GAS (n = 3584/5013, 71.5%). The predominant masculinizing GAS observed was metoidioplasty, accounting for 63.3% (n = 2270/3584) of cases.

It is noteworthy, however, that these volumes are driven by data from two specific institutions in Serbia, which performed a total of 1751 metoidioplasties, accounting for nearly half (48.9%) of all masculinizing GAS [24,37,38,48,50]. Serbia reported the highest incidence of masculinizing GAS (n = 3126/3584, 87.2%) overall. Gender-affirming hysterectomy was the second most prevalent masculinizing GAS (n = 1214/3584, 33.9%), followed by phalloplasty (n = 813/3584, 22.7%). Hysterectomy volumes were largely driven by Serbia, with one of the same high-volume Serbian institutions performing metoidioplasty (n = 593/1214, 48.8%), as well as India (n = 558/1214, 46%). Phalloplasty volumes were predominantly reported by India (n = 552/813, 67.9%), where 82.5% (n = 671/813) of cases utilized free radial artery forearm flap and the remainder employed the pedicled anterolateral thigh flap (n = 142/81, 17.5%). Table 2A shows the distribution of the different masculinizing GAS by country.

Table 2B reports the frequency of post-operative complications following masculinizing GAS procedures. Of 3584 patients who underwent masculinizing GAS, 11.6% (n = 416/3584) experienced at least one complication and 7.2% (n = 258/3584) required revision. The most common complications included perineal cysts (n = 79/2270, 3.5%) following metoidioplasty and urethral strictures following phalloplasty (n = 145/813, 17.8%).

Although chest masculinization surgery remains the most common masculinizing surgery in the United States, it was the fourth most commonly reported masculinizing GAS in LMICs comprising only 13.1% of patients (n = 471/3584).

#### 3.3.2. Feminizing GAS

While 55.8% (n = 19/34) of studies [16,17,19,20,22,23,26,27,28,29,31,32,33,35,43,44,45,46] included patients who underwent feminizing GAS, the total number of TGNB individuals who underwent feminizing GAS (n = 1480/5064, 29.2%) was significantly lower than of those who underwent masculinizing GAS. The distribution of the feminizing GAS procedures and their respective incidences across LMICs are shown in Table 3A.

Vaginoplasty was the most common feminizing GAS, representing 74.5% (n = 1103/1480) of reported cases, and was predominantly performed in India (n = 685/1103, 62.1%), followed by Brazil (n = 249/1103, 22.6%). Among n = 1103 patients, approximately half (n = 580/1103, 52.6%) underwent a form of penile inversion vaginoplasty, using a classic or penile-perineoscrotal flap approach [19,22,27,35,46]. The remainder (n = 395/1103, 35.8%) largely underwent the modified rectosigmoid colon approach for vaginoplasty [20,33]. A total of 4.7% (n = 52/1103) of patients reported undergoing peritoneal pull-through vaginoplasty [28], and 3.2% (n = 35/1103) reported secondary vaginoplasty using grafts derived from abdominal tissue [45]. One study comprising n = 41 patients did not disclose the approach utilized for vaginoplasty [43].

Facial feminizing surgery accounted for 16.5% (n = 244/1480) of feminizing GAS, with India reporting the highest volume (n = 227/244, 93%), followed by Iran (n = 10/244, 4.1%). Chondrolaryngoplasty comprised 4.4% (n = 65/1480) of cases and was predominantly conducted in India (n = 55/65, 84.6%) and Iran (n = 10, 15.4%).

Table 3B reports the frequency of post-operative complications following feminizing GAS procedures.

Of n = 1480 patients who underwent feminizing GAS, n = 185/1480 (12.5%) experienced at least one complication, and n = 97/1480 (6.6%) required revision surgery. In patients who underwent vaginoplasty (n = 1103), complications most commonly included the development of granulomatous tissue (n = 44/1103, 4%) and urethral strictures (n = 44/1103, 4%). The most common complication following facial feminizing surgery was wound dehiscence (n = 5/244, 2.1%). This was also the case for chondrolaryngoplasty (n = 4/65, 6.2%).

### 3.4. Patient Satisfaction and Quality of Life Outcomes

A total of 25/34 (73.5%) studies with n = 3243 patients reported on post-operative satisfaction and quality of life metrics [16,20,21,22,23,24,25,27,28,29,31,32,35,37,38,40,41,42,43,44,45,48,49]. This was markedly heterogenous, with different methodologies for assessing quality of life. While few studies formally utilized validated scoring systems, such as the WHO Quality of Life Brief Questionnaire, PHQ-9, and the Rosenberg self-esteem scale, the majority applied subjective patient perception questionnaires during follow-up. These aimed to determine whether patients perceived an improvement in their quality of life. Satisfaction was assessed heterogeneously across all studies. Patients were asked to self-assess their satisfaction using categorical scales ranging from ‘unsatisfactory’ to ‘very high satisfaction’ across various parameters, including aesthetic outcome, erogenous sensation, sexual function, vocal change, or an overall assessment relevant to the procedure.

While it is difficult to amalgamate outcomes across all studies reviewed, several significant findings emerged. The study by Castanon et al. [28] in Serbia highlighted high patient satisfaction following peritoneal pull-through vaginoplasty, with 96% of patients expressing satisfaction with both appearance and sensitivity. Ozkan et al. [20] utilized a rectosigmoid colon-based approach for vaginal reconstruction, reporting favorable outcomes in both aesthetics and sexual functioning. Bordas et al. reported high satisfaction among 645 patients regarding post-operative aesthetics and erogenous sensation following metoidioplasty [24]. While only 17.6% (6/34) of studies with a total of n = 343 patients specifically evaluated post-operative quality of life, nearly all studies indicated statistically significant improvements across most domains and cohorts (Table 1). For example, in the study by Sir et al., 71 mastectomy patients used a validated TRANS-Q PROM. There was a significant improvement in satisfaction between pre-operative and post-operative assessments (*p* < 0.05) across cohorts for each specific mastectomy approach [42]. Likewise, the study conducted by Chaovanilikit et al. in Thailand, involving 37 vaginoplasty patients, revealed that surgery led to significant improvements in quality of life, depression, and self-esteem (*p* < 0.001) [43].

Despite overall promising outcomes, several limitations remain. In their series of 160 phalloplasty cases, Bencic et al. reported only a 14% satisfaction rate for tactile sensation in the neophallus [25]. Furthermore, across the 41 vaginoplasty cases included in the de Toni Abboud et al. study in Brazil [45], 22.9% of patients were dissatisfied with cosmesis and sexual function. They also reported concerns regarding satisfactory sexual intercourse and neovaginal length. Similarly, relatively high rates of sexual dissatisfaction were reported by 26.9% of patients post-vaginoplasty in the Monteiro Petry Jardim study, also conducted in Brazil [44]. Sexual function consistently emerged as the least satisfactory domain in 7 out of 8 studies where it was assessed [20,28,30,35,38,44,45,48]. The heterogeneity of reporting makes it difficult to assess other domains beyond sexual function [16,20,21,22,23,24,25,27,28,29,30,31,32,35,37,38,40,41,42,43,44,45,48,49].

Of 216 patients surveyed for post-surgical regret, none expressed regret [16,20,21,22,23,24,25,27,28,29,31,32,35,37,38,40,41,42,43,44,45,48,49]. Regret was subjectively assessed through patient follow-up questionnaires where applicable. Among studies that assessed quality of life, satisfaction, or post-operative regret, there was no standardized questionnaire used for assessing these outcomes.

## 4. Discussion

This systematic review, following PRISMA and Cochrane guidelines, highlights geographic disparities in GAS literature, with 35.3% of the literature originating from lower-middle-income countries and no studies from low-income countries. Masculinizing GAS, primarily metoidioplasty, constituted 70.8% of cases, notably influenced by Serbian outcomes [24], while feminizing GAS, mainly vaginoplasty, accounted for 29.2% of surgeries. The rate of post-operative complications for most procedures was within reported ranges in the general literature. Patient satisfaction across both masculinizing and feminizing surgeries was high, although no validated or standardized questionnaires were used for assessment, and heterogeneity in outcomes was observed. The review provides a preliminary understanding of GAS outcomes in LMICs, emphasizing geographical variations, prevalent procedures, complications, and patient satisfaction outcomes.

Our findings highlight a global increase in GAS reporting. Notably, nearly half of the included studies were published in the two years preceding the review (n = 14 [41.1%]). This trend aligns with existing evidence pointing to the increasing prevalence of GAS on a global scale [51,52]. In contrast, reporting of genital surgery was more prevalent than chest surgery in both masculinizing and feminizing gender-affirming procedures. This differs from findings from the Nationwide Ambulatory Surgery Sample and the National Inpatient Sample in the United States, where breast and chest GASs were reported as the most common procedures, followed by genital reconstruction [53]. It is unclear if this inconsistency arises from bias in reporting or is an actual reflection of overall surgical volumes. The rise of medical tourism in countries such as Thailand [54] and Serbia [55] may lead to an increased volume of complex GAS being performed in certain LMICs. Specifically, in cases where GAS procedures are not covered by insurance, patients may opt to self-finance less complex procedures, such as breast augmentation or chest masculinization, in their home countries. However, they may choose to travel abroad, particularly for more affordable vaginoplasty, metoidioplasty, and phalloplasty procedures.

In our review, the reporting of post-operative complications was inconsistent, resulting in a wide range of outcomes. This inconsistency aligns with global data, which also faces challenges with inconsistent reporting of outcome parameters [56]. Vaginoplasty was the most common feminizing procedure in this review, and complications such as urethral strictures and granulomatous tissue development were low, occurring in less than 5% of cases, a notably lower incidence compared to reports from HICs, where vaginoplasty complications range from 20–30% [57,58]. We postulate that this discrepancy may stem from challenges to follow-up, insufficient patient monitoring due to poor access to healthcare services, lack of standardization in outcome measurements, and controversy in what constitutes a complication. Furthermore, this inconsistency may be driven by socioeconomic disparities within LMICs. Chaovanilikit suggests that TGNB patients who successfully access GAS in LMICs are wealthier and have better access to safe recovery environments [43], both of which are known to be positive predictors of GAS outcomes [51]. This contrasts with the GAS population in HICs, which can be comprised of a more socioeconomically diverse population. Lastly, as highlighted by Thammapiwan [27], studies from LMICs may exhibit more methodological flaws and smaller sample sizes compared to studies conducted in HICs. This discrepancy is often exacerbated by the availability of greater academic and research resources, as well as higher-volume surgical facilities in HICs [22].

Metoidioplasty, the most prevalent masculinizing GAS in this review, represented 63.3% of cases, primarily from the 813 patients included in the study by Bordas et al. in Serbia [24]. As the literature is heavily influenced by Serbian centers performing high volumes of metoidioplasty, it is hard to ascertain the true prevalence of masculinizing genital surgery. Nonetheless, many reconstructive centers in LMICs may lack the necessary infrastructure and expertise for microsurgically based phalloplasty. Hence, metoidioplasty may be considered a more feasible option in such settings. Lastly, it is not uncommon for patients to elect for single stage metoidioplasty as opposed to multistage phalloplasty, due to financial limitations, and concerns for high rates of complications associated with phalloplasty.

This review underscores the critical importance of employing a standardized questionnaire to assess satisfaction and quality of life among TGNB individuals undergoing GAS that can be universally applied in both HICs and LMICs. The goal is to establish a common metric that transcends geographical and cultural variations, ensuring that assessments of satisfaction and quality of life are applicable and comparable across diverse settings. By addressing this need for standardization, future research can contribute significantly to enhancing the consistency and reliability of data on the outcomes of GAS, ultimately advancing the understanding of the impact of these procedures on the well-being of individuals across different socioeconomic and cultural contexts.

The overall paucity of studies in this review again highlights the unfortunate reality that GAS remains a significant barrier for TGNB individuals in LMICs. In such settings, many patients seeking GAS opt for practices that may diverge from international or country-based standards of care due to stigma and legal constraints. Majumder et al. highlights that up to 13.6% of patients in Eastern India received GAS outside the standards of care [18,19] and a small minority received services by actual licensed medical practitioners. Similarly, Bautista et al. stress the lack of trained providers in Colombia, as well as the high proportion of patients who engage in medical transition without a prescription or medical supervision. They highlight that their findings are consistent with reports from Thailand, another upper-middle income country, where 88.6% of TGNB patients acknowledged self-administering hormones [26,27,43].

Several studies emphasized the persistent social challenges faced by TGNB individuals in LMICs. Aghabikloo et al. [17] commented on the unique interplay between society-level stigma and regulatory approval for GAS in Iran. They noted that the suicide rate among TGNB individuals in Iran exceeds that of the general population (20% vs. 0.006%). Additionally, GAS is only sanctioned following successful fulfilment of selection criteria, which include, among other factors, parental consent. Due to their marginalized status and resulting economic challenges within LMICs, TGNB individuals often face significant financial constraints. The substantial cost associated with GAS can therefore serve as a formidable barrier to surgical access for this population. A scoping review conducted in 2020 by Scheim et al. [8] highlighted peer and family rejection, along with financial obstacles, as regrettably common experiences for TGNB individuals in LMICs seeking GAS. This was supported by Barik et al., who emphasized the substantial financial exclusion experienced by the TGNB community in India [59]. In fact, India stands as the sole country in this review with any evidence of publicly-funded access to GAS [59]. Given the disadvantaged economic status of TGNB individuals in LMICs, the expenses associated with GAS may pose a particularly significant barrier for this population [60,61,62].

Our study should be interpreted in light of some limitations. First, only 47.1% [16,17,20,21,22,23,29,31] of studies reported on patient demographics and pre-operative characteristics. Second, overall complication rates (Table 2B and Table 3B) were computed by dividing the number of observed complications by the total number of patients in studies where the complication was assessed. However, because not all studies assess for or report these complications, the reported rates may significantly underestimate the true complication rates. This discrepancy is primarily attributed to the limitations inherent to the included studies and their methodologies. In addition, many of the studies originated from countries where TGNB individuals face persecution, and access to GAS, where available, is not formally documented. This may have contributed to publication bias. The focus on English language studies in the inclusion criteria might have inadvertently excluded relevant studies from LMICs where English is not the primary language. Our review included studies with varying sample sizes, with only five (14.7%) studies involving samples larger than 250 individuals [24,30,33,34,48], underscoring the need for larger international studies on GAS. Lastly, the studies had predominantly short follow-ups, with only two studies extending beyond 5 years, impeding the comprehensive assessment of long-term outcomes and potential complications. Despite these considerations, efforts were made to address these limitations within the bounds of the review process.

## 5. Conclusions

We observed increased reporting of GAS outcomes in LMICs in recent years, and several studies highlighted significant quality of life improvements following GAS. Despite these findings, LMICs face significant challenges due to limited infrastructure, restricted access to care, and enduring stigma impacting the journey of TGNB individuals. The limited and heterogeneous outcomes reported in the literature underscore the need for additional research, focusing on standardized metrics for capturing long-term follow-up, patient satisfaction, and quality of life outcomes. International collaboration is crucial, fostering shared responsibility to promote global surgical equity by supporting provider training and knowledge exchange between high-income and LMIC settings.

## Figures and Tables

**Table 1:** Characteristics of Studies Included.

Lead Author, Year of Publication	Country	Study Design	Mean Age, Years	Surgeries (No.)	Complications (%)	No. (%) of Patients with at Least One Complication	Domains Assessed for Post-Operative Satisfaction (% Patients with Improvement)	Mean Follow-Up (Months)
Koak. 2010 [16]	Turkey ^δ^	Case series, multi-center	29.2	Laser reduction glottoplasty (6)	Edema (100%)	6 (100%)	Voice quality (100%) Voice-related QOL showed significant improvement	13.4
Aghabikloo, 2012 [17]	Iran ^Φ^	Cross-sectional, single center	24.9	Feminizing surgery (44) Masculinizing surgery (25)	NA	NA	NA	NA
Majumder, 2016 [18]	India ^Φ^	Retrospective cohort, single center	NA	Mastectomy (9) Hysterectomy (7) Salpingo-oopherectomy (7) Phalloplasty (1)	NR	NR	NA	19.2
Majumder, 2017 [19]	India ^Φ^	Retrospective cohort, single center	25.8	Breast augmentation (13) Orchiectomy (14)	NR	NR	NA	22.8
Ozkan, 2018 [20]	Turkey ^δ^	Retrospective cohort, single center	40	Vaginoplasty (9)	Hematoma (2%)	2 (22.2%)	Aesthetics (100%) Sexual function (93.3%)	37.2
Naeimi, 2019 [21]	Iran ^Φ^	Prospective cohort, single center	34.2	Masculinizing surgery (42)	NA	NA	Mean QoL of total and all other domains show significant improvement using validated PROM	6
Moises da Silva, 2021 [22]	Brazil ^δ^	Retrospective cohort, single center	32.2	Vaginoplasty (216)	Granulation tissue formation (20.5%) Urethral meatus stricture (20.5%) Introital stricture (15.4%) Wound dehiscence (12.6%) Hematoma/excessive bleeding (8.9%) Need for transfusion (7.9%) Tissue necrosis (1.9%) Urethral fistula (1.9%) Intra-operative rectal injury (1.9%) Rectovaginal fistula (0.9%)	82 (44%)	Sexual function (85%)	16
Aires, 2021 [23]	Brazil ^δ^	Prospective cohort, multi-center	35.4	Glottoplasty (7)	Wound dehiscence (14.3%) Granulation Tissue Formation (14.3%)	2 (28.6%)	Voice-related QoL showed significant improvement	13.7
Bordas, 2021 [24]	Serbia ^δ^	Retrospective cohort, single center	24.4	Metoidioplasty ^Γ^ (813) Hysterectomy (156) Mastectomy (58)	Urethral stricture ^Γ^ (55%) Urethral fistula ^Γ^ (10%) Vaginal remnant ^Γ^ (9.6%) Testicular displacement ^Γ^ (3.2%) Testicular implant rejection ^Γ^ (2%)	207 (25.5%)	Tactile and erogenous sensation ^Γ^ (100%) Aesthetics ^Γ^ (99%)	94
Bencic, 2022 [25]	Serbia ^δ^	Case series, single center	NA	Phalloplasty (160)	Urethral fistula (25%) Implant rejection (3.8%) Surgical site infection (3.8%) Urethral stricture (2.5%) Flap necrosis (1.9%)	NA	Aesthetics (100%) Erogenous sensation (100%) Tactile sensation (14.4%)	52
Bautista, 2022 [26]	Colombia ^δ^	Case series, single center	NA	Mastectomy (3) Vaginoplasty (2) Augmentation mammaplasty (2)	NA	NA	NA	NA
Thammapiwan, 2022 [27]	Thailand ^δ^	Cross sectional, multi-center	29.3	Vaginoplasty (69)	NA	NA	QoL (97.1%) Sexual function (17.4%)	NA
Castanon, 2022 [28]	Serbia ^δ^	Case series, single center	27	Vaginoplasty (52)	Hematoma (5.8%) Superficial necrosis (5.8%) Wound dehiscence (5.8%)	7 (13.5%)	Sensitivity (96%) Lubrication (96%) Sexual function (96%)	29
Balaji, 2016 [29]	India ^Φ^	Retrospective cohort, single center	26.3	Facial feminizing surgery (7)	NA	0 (0%)	Overall satisfaction (100%)	NA
Stojanovic, 2017 [30]	Serbia ^δ^	Retrospective cohort, single center	31.5	Metoidioplasty ^Γ^ (473) Hysterectomy ^Λ^ (137) One-stage GAS * (79)	Perineal cyst * (11.5%) Urethral fistula * (5.1%) Urethral stricture * (3.8%) Testicular implant rejection * (3.8%) Urethral diverticulum * (1.3%) Nipple graft necrosis * (3.8%) Breast hematoma * (1.3%)	20 (25.3%)	Voiding * (100%) Erection * (100%) Erogenous sensation * (100%) Aesthetics * (96.2%) Sexual function * (87.3%)	44
Telang, 2020 [31]	India ^Φ^	Retrospective cohort, single center	38	Facial Feminization Surgery (220)	Minor wound dehiscence (1.8%) Contour irregularities (0.9%) Hematoma (0.5%)	8 (3.6%)	Overall Satisfaction (100%)	40
Shams, 2009 [32]	Iran ^Φ^	Case series, single center	23	Facial Feminization Surgery (10)	NA	NA	Aesthetics (100%)	NA
Kaushik, 2019 [33]	India ^Φ^	Retrospective cohort, single center	39	Vaginoplasty (386)	Mucorrhoea (6.2%) Mucosal Prolapse (1.5%) Dyspareunia (1.5%) Stricture introitus (1.5%)Urinary Retention (1.3%) Paralytic Ileus (0.8%) Wound Infection (0.8%) Colon Graft Loss (0.5%) Clitoral Necrosis (0.5%)	78 (20.2%)	Overall Satisfaction (94%)	34
Gupta, 2022 [34]	India ^Φ^	Retrospective cohort, single center	NA	Phalloplasty (551)	Urethral Stricture (22%) Urethral Fistula (9.8%)	121 (22%)	NA	NA
Gupta, 2022 [35]	India ^Φ^	Retrospective cohort, single center	29	Vaginoplasty (183)	NA	NA	Sensitivity (100%) Sexual function (76%)	31
Gao, 2022 [36]	China ^δ^	Retrospective cohort, single center	25.3	Phalloplasty (80)	Urethral Fistula (35%) Flap necrosis (32.5%) Urethral Stricture (25%)	28 (35%)	Satisfaction with voiding was high, with no significant difference between phalloplasty approaches; QoL improved, with significant differences between the traditional and modified approaches	6.1
Djordjevic, 2009 [37]	Serbia ^δ^	Prospective cohort, single center	32	Metoidioplasty (38)	Urethral Fistula (5.3%) Urethral Erosion (2.6%)	3 (7.9%)	Aesthetics (100%)	22
Vukadinovic, 2014 [38]	Serbia ^δ^	Prospective cohort, single center	29	Metoidioplasty (97)	Urethral Fistula (6.2%) Urethral Stricture (2.1%) Testicular Displacement (2.1%)	27 (27.8%)	Voiding (100%) Erection (100%) Erogenous Sensation (100%) Aesthetics (95.9%) Sexual Function (87.6%)	30
Gupta, 2017 [39]	India ^Φ^	Retrospective cohort, single center	NA	Mastectomy (20)	NA	NA	NA	NA
Jeftovic, 2018 [40]	Serbia ^δ^	Retrospective cohort, single center	28.5	Hysterectomy (124)	NA	2 (1.6%)	NA	41
Gumussoy, 2022 [41]	Turkey ^δ^	Prospective cohort, single center	27.3	Mastectomy (66) Hysterectomy (66)	NA	NA	NA	6
Sir, 2022 [42]	Turkey ^δ^	Prospective cohort, single center	25	Mastectomy (71)	NA	NA	Overall satisfaction using validated PROM	NA
Chaovanalikit, 2022 [43]	Thailand ^δ^	Prospective cohort, single center	26.2	Vaginoplasty (37)	NA	0 (0%)	QoL and self-esteem showed significant improvement;All patients with pre-operative depression (13.5%) reported no depression post-operatively	NA
Monteiro Petry Jardim, 2022 [44]	Brazil ^δ^	Retrospective cohort, single center	39.9	Vaginoplasty (26)	Rectovaginal Fistula (3.8%)	7 (26.9%)	Overall satisfaction (84.6%) Sexual function (73.1%) Improvement in QoL across all domains was high (range: 72.9% to 93.7%) using validated PROM	NA
de Toni Abboud, 2022 [45]	Brazil ^δ^	Retrospective cohort, single center	41	Vaginoplasty (35)	Introital stenosis (22.9%) Rectal Fistula (8.6%) Wound Dehiscence (2.9%) Urethral fistula (2.9%)	10 (28.6%)	Overall satisfaction (77.1%) Sexual function (77.1%)	62
Kumar, 2023 [46]	India ^Φ^	Prospective cohort, single center	29	Vaginoplasty (100)	Bleeding (4.8%)	1 (4.8%)	Sexual function (76%)	11
Cheng, 2021 [47]	China ^δ^	Retrospective cohort, single center	25.6	Phalloplasty (21)	Urethral Fistula (52.4%) Urethral Stricture (5.8%)	12 (57.1%)	Aesthetics-phallus appearance (76.2%) Aesthetics-scar (81%)	12
Stojanovic, 2023 [48]	Serbia ^δ^	Retrospective cohort, single center	29	Metoidioplasty (938)	Urethral Fistula (12.3%) Perineal cyst (9.8%) Implant rejection (5.9%) Urethral Stricture (2.6%) Diverticulum (1.7%)	143 (15.2%)	Sexual Function (100%) Voiding (99%)	NA
Top, 2017 [49]	Turkey ^δ^	Prospective cohort, single center	28.2	Mastectomy (52)	Superficial necrosis (9.6%) Hematoma (3.8%)	7 (13.4%)	Aesthetics (96%)	28

Acronyms: QoL: Quality of Life, No.: number, NR: Not Reported, NA: Not Applicable. Type of surgery—^Γ^: Metoidioplasty, ^Λ^: Hysterectomy; Country classification—^δ^: Upper-middle income country, ^Φ^: Lower-middle income country. *: One-stage GAS (chest masculinization, total transvaginal hysterectomy with bilateral adnexectomy, vaginectomy, metoidioplasty, urethral lengthening, scrotoplasty, and implantation of bilateral testicular prostheses).

**Table 2:** (**A**). Global Distribution and Incidence of Masculinizing Gender-Affirming Surgery Procedures. (**B**). Frequency of Complications Following Masculinizing Gender-Affirming Surgery Procedures.

**(A)**		
**Procedure**	**Incidence (n = 3584)**	**Countries (in Order of Volume)**
Metoidioplasty	63.3% (n = 2270)	Serbia (n = 2270) [24,30]
Hysterectomy	33.9% (n = 1214)	Serbia (n = 593) [18,34] India (n = 558) [18,34]
Phalloplasty	22.7% (n = 813)	India (n = 552) [18,34] Serbia (n = 160) [25] China (n = 101) [36]
Chest masculinizing surgery	13.1% (n = 471)	Serbia (n = 261) [24,25] Turkey (n = 186) [41,42,49] India (n = 29) [18]
**(B)**		
**Procedure**	**Complication**	**Incidence**
Metoidioplasty (n = 2270)	Perineal cyst	3.5% (n = 79) [24,25]
	Urethral fistula	3.3% (n = 76) [24,25]
	Urethral stricture	0.9% (n = 20) [24,25]
Hysterectomy (n = 1214) and Phalloplasty (n = 813)	Urethral stricture	14% (n = 283) [18,24,30,34]
	Urethral fistula	12.4% (n = 252) [18,24,30,34]
	Implant rejection	0.9% (n = 20) [18,24,30,34]
	Flap necrosis	2.1% (n = 42) [18,25,34,36]

N.B.: Complication rates were computed by dividing the number of observed complications by the total number of patients in studies where the complication occurred. Reported rates may underestimate the true complication rates.

**Table 3:** (**A**). Global Distribution and Incidence of Feminizing Gender-Affirming Surgery Procedures. (**B**). Frequency of Complications Following Feminizing Gender-Affirming Surgery Procedures.

**(A)**		
**Procedure**	**Incidence (n = 1273)**	**Countries (in Order of Volume)**
Vaginoplasty	74.5% (n = 1103)	India (n = 685) [19,33,35] Brazil (n = 249) [22] Thailand (n = 110) [27] Serbia (n = 52) [28] Turkey (n = 9) [20]
Facial feminizing surgery	16.5% (n = 244)	India (n = 227) [29,31] Iran (n = 10) [32] Brazil (n = 7) [23]
Thyrochondroplasty	4.4% (n = 65)	India (n = 55) [31] Iran (n = 10) [32]
Breast augmentation	1% (n = 13)	India (n = 13) [19]
**(B)**		
**Procedure**	**Complication**	**Incidence**
Vaginoplasty (n = 1103)	Development of granulomatous tissue	3.9% (n = 44) [22,23]
	Urethral stricture	3.9% (n = 44) [19,20,22,27,28,33,35]
	Introital stricture	3.5% (n = 39) [19,22,27,33,35]
Facial feminizing surgery (n = 244)	Wound dehiscence	2.1% (n = 5) [29,31,32]
	Contour irregularities	0.8% (n = 2) [29,31]
Thyrochondroplasty (n = 65)	Wound dehiscence	6.2% (n = 4) [31,32]
	Contour irregularities	3.1% (n = 2) [31,32]

N.B.: Complication rates were computed by dividing the number of observed complications by the total number of patients in studies where the complication occurred. Reported rates may underestimate the true complication rates.

## Supplementary Materials

The following supporting information can be downloaded at: https://www.mdpi.com/article/10.3390/jcm13123580/s1, Document S1: Search Terms. Document S2: List of LMICs included in this review, as per the World Bank’s 2023 GNI per capita classifications.
