# Supplementary material for: Gender-Affirming Surgery in Low- and Middle-Income Countries: A Systematic Review
Source: J Clin Med. 2024 Jun 19;13(12):3580. doi: 10.3390/jcm13123580 (PMC11205133; doi:10.3390/jcm13123580)
Supplement: Supplementary file 1 [file jcm-13-03580-s001.zip › jcm-3001286-Document S1.pdf]

Document S1. Search Terms.

1. ((chest surgery or genital surgery or chest masculinising surgery or chest masculinizing surgery or chest feminization surgery or chest feminisation surgery or chest masculiniz* or chest feminiz* or chest masculinis* or chest feminis* or mammoplasty or mammaplasty or vaginoplasty or vulvoplasty or phalloplasty or penile prosthesis surgery or facial surgery or facial feminization surgery or facial feminisation surgery or facial masculinization surgery or facial masculinisation surgery or facial masculiniz* or facial feminiz* or facial masculinis* or facial feminis* or facial surgery transgender or facial surgery trans or genital reassignment surgery or voice feminisation surgery or voice masculinisation surgery or voice feminization surgery or voice masculinization surgery or voice feminiz* or voice masculiniz* or voice feminis* or voice masculinis* or glottoplasty or orchiectomy or face femini* or facial femini* or forehead femini* or frontal bone surgery or cheek femini* or chin femini* or eye femini* or eyelid femini* or eyebrow femini* or nasal femini* or jaw femini* or nasolabial fold femini* or face masculini* or facial masculini * or forehead masculini * or cheek masculini* or chin masculini* or eye masculini* or eyelid masculini* or eyebrow masculini* or nasal masculini* or jaw masculini* or nasolabial fold masculini* or masculini* surgery or femini* surgery or chest surgery or genital surgery or

chest masculinis* surgery or chest masculiniz* surgery or chest feminiz* surgery or chest feminis* surgery or breast augment* or breast reduct* or chondrolaryngoplast* or feminiz* nasal surgery or masculiniz* nasal or feminis* nasal or masculinis* nasal or neck rhinoplast* or mammoplasty or mammaplasty or orchiectomy or tracheal shave or penectomy or metiodioplasty or mastectomy or vaginoplasty or vulvoplasty or phalloplasty or penile prosthesis surgery or facial surgery or facial feminization surgery or facial feminisation surgery or facial masculinization surgery or facial masculinisation surgery or facial surgery transgender or facial surgery trans or genital reassignment surgery or voice feminisation surgery or voice masculinisation surgery or voice feminization surgery or voice masculinization surgery or glottoplasty or orchiectomy or chondroplast* or neck rhinoplast* or mandibular reconstruct* or mandibular setback or Frontal sinus setback or Forehead setback or Rhinoplasty or Top surgery or Gonial angle or Genioplasty or Shallow depth vaginoplasty or Zero depth vaginoplasty or Vocal surgery or Urethral lengthening or Penile implant or Scrotoplasty or Penile reconstructi* or Vaginal reconstructi*) and (Transgender Persons or Transsexualism or Gender Identity or transsexual or transgender or gender identity or trans-gender or trans-sexual or gender

identity or gender identification or gender identified or transperson or sexual dysphoria or cross-gender or cross gender or feminiz* or masculiniz* or feminis* or masculinis* or gender congruence surgery or gender modif* surgery or top surg* or bottom surg* or Transgender Persons or Transsexualism or Gender Identity or transsexual or transgender or gender identity or trans-gender or trans-sexual or gender identity or gender identification or gender identified or transperson or sexual dysphoria or cross-gender or cross gender or feminiz* or masculiniz* or Travesty or transexual or FTM or MTF or male to female transgender or female to male transgender or koti or hijra or mahu or waria or katoey or cross dresser or transvestite or transvestites or transman or transwoman or transmen or transwomen))).mp. [mp=ti, ab, hw, tn, ot, dm, mf, dv, kf, fx, dq, cw, bt, nm, ox, px, rx, ui, sy]

2. (LMIC or Low Income or Middle Income or low-middle income or Afghanistan or Benin or Burkina Faso or Burundi or Central African Republic or Chad or Democratic Republic of the Congo or Eritrea or Ethiopia or Gambia or Guinea or Guinea-Bissau or Haiti or Democratic People's Republic of Korea or Liberia or Madagascar or Malawi or Mali or Mozambique or Nepal or Niger or Rwanda or Sierra Leone or Somalia or

South Sudan or Syria or Tajikistan or Tanzania or Togo or Uganda or Yemen).ab,in,ti.

3.	Developing Countries.af.
4.	(Low* income* adj3 (countr* or nation* or economy or economies)).tw.
5.	(LIC* adj3 (countr* or nation* or economy or economies)).tw.
6.	((Developing or "under developed" or underdeveloped or less- developed or "less* developed" or "third world") adj3 (countr* or nation* or economy or economies)).tw.
7.	"transition* countr*".tw.
8.	(Afghan* or Benin* or "Burkina Faso" or Burundi* or "Central African Republic" or Chad* or "Democratic Republic of the Congo" or Eritrea* or Ethiopia*).ab,in,ti.
9.	((Underserved or "under served" or deprived or poor*) adj3 (countr* or nation* or economy or economies)).tw.
10.	(Gambia* or Guinea* or Guinea-Bissau* or Haiti* or "North Korea*" or "DPR Korea*" or "Korea* DPR" or "Democratic people* republic of Korea*" or "Korea* democratic people* republic" or "DPRK").ab,in,ti.

11. (Liberia* or Madagasca* or Malawi* or Mali* or Mozambique* or Nepal* or Niger or Rwanda*).ab,in,ti.
12. ("Sierra Leone" or Somalia* or "South Sudan*" or Syria* or "Syrian Arab Republic" or Tajikistan* or Tanzania* or Togo or Uganda* or Yemen*).ab,in,ti.
13. (Low-middle income* adj3 (countr* or nation* or economy or economies)).tw.
14. (Lower-middle income* adj3 (countr* or nation* or economy or economies)).tw.
15. (LMIC* adj3 (countr* or nation* or economy or economies)).tw.
16. LMIC*.tw.

17. (Angola or Bangladesh or Bhutan or Bolivia or Cabo Verde or Cambodia or Cameroon or Comoros or Congo or Cote d'Ivoire or Djibouti or Egypt or El Salvador or Ghana or Honduras or India or Indonesia or Kenya or Kyrgyzstan or Laos or Lesotho or Mauritania or Micronesia or Moldova or Mongolia or Morocco or Myanmar or Nicaragua or Nigeria or Pakistan or Papua New Guinea or Philippines or Atlantic Islands or Melanesia or Senegal or Sudan or Swaziland or Timor-Leste or Tunisia or Ukraine or Uzbekistan or Vanuatu or Vietnam or Zambia or Zimbabwe).ab,in,ti.
18. (Angola* or Bangladesh* or Bhutan* or Bolivia* or "Cabo Verde" or Cambodia* or Cameroon* or Comoros* or Congo or "Cote D'Ivoire" or "Ivory Coast" or Djibouti* or Egypt* or "El Salvador" or Ghana* or Honduras*).ab,in,ti.
19. (India* not "american indian*").ab,in,ti.
20. (Indonesia* or Kenya* or Kiribati* or Kyrgyzstan* or "Kyrygyz Republic" or Lao or Laos or Lesotho or Mauritania* or Micronesia* or Moldova* or Mongolia* or Morocco* or Moroccan* or Myanmar* or Burma or Burmese).ab,in,ti.

21. (Nicaragua* or Nigeria* or Pakistan* or "Papua New Guinea*" or Philippines or Filipino or "Sao Tome and Principe" or Senegal* or "Solomon Islands").ab,in,ti.

22. ((Sudan* not "South Sudan*") or Swaziland* or Eswatini* or Timor* or Tunisia* or Ukrain* or Uzbekistan* or Vanuatu* or Vietnam* or "West Bank" or Gaza or Zambia* or Zimbabw*).ab,in,ti.

23. (Upper-middle income* adj3 (countr* or nation* or economy or economies)).tw.

24. (UMIC* adj3 (countr* or nation* or economy or economies)).tw.

25. (Middle income* adj3 (countr* or nation* or economy or economies)).tw.

26. (Low adj2 middle income* adj3 (countr* or nation* or economy or economies)).tw.

27. (Albania or Algeria or American Samoa or Argentina or Armenia or Azerbaijan or "Republic of Belarus" or Belize or "Bosnia and Herzegovina" or Botswana or Brazil or Bulgaria or China or Colombia or Costa Rica or Cuba or Dominica or Dominican Republic or Equatorial Guinea or Ecuador or Fiji or Gabon or "Georgia (Republic)" or Grenada or Guatemala or Guyana or

Iran or Iraq or Jamaica or Jordan or Kazakhstan or Kosovo or Lebanon or Libya or "Macedonia (Republic)" or Malaysia or Indian Ocean Islands or Mauritius or Mexico or Montenegro or Namibia or Paraguay or Peru or Romania or Russia or Samoa or "Independent State of Samoa" or Serbia or Sri Lanka or South Africa or Saint Lucia or "Saint Vincent and the Grenadines" or Suriname or Thailand or Tonga or Turkey or Turkmenistan or Venezuela).ab,in,ti.

28. (Albania* or Algeria* or "American Samoa*" or Argentina* or Armenia* or Azerbaijan* or Belarus* or Belize* or Bosnia* or "Bosnia adj2 Herzegovina*" or Botswana* or Brazil* or Bulgaria* or China* or Chinese or Columbia* or "Costa Rica*" or Cuba* or Dominica* or "Dominican Republic" or "Equatorial Guinea*").ab,in,ti.

29. (Ecuador* or Fiji* or Gabon* or Georgia* or Grenada* or Guatemala* or Guyana* or Guyanese or Iran* or Persia* or Iraq* or Jamaica* or Jordan* or Kazakhstan* or Kosovo* or Lebanon* or Lebanese or Libya* or Macedonia* or Malaysia* or Maldives or "Marshall Islands" or Mauritius* or Mauritian or Mexic*).ab,in,ti.

30. (Montenegro* or Namibia* or Nauru* or Paraguay* or Peru* or Romania* or Russia* or Samoa* or Serbia* or "South Africa*" or "Sri Lanka*" or "St Lucia*" or "Saint Lucia*" or "St Vincent adj3 Grenadines" or "Saint Vincent adj3 Grenadines").ab,in,ti.
31. (Suriname* or Thai* or Tonga* or Turkey* or Turkish or Turkmenistan* or Tuvalu* or Venezuela*).ab,in,ti.
32. 2 or 3 or 4 or 5 or 6 or 7 or 8 or 9 or 10 or 11 or 12 or 13 or 14 or 15 or 16 or 17 or 18 or 19 or 20 or 21 or 22 or 23 or 24 or 25 or 26 or 27 or 28 or 29 or 30 or 31
33. 1 and 32
34. limit 33 to english language
