# Supplementary material for: Gender-Affirming Surgery in Low- and Middle-Income Countries: A Systematic Review
Source: J Clin Med. 2024 Jun 19;13(12):3580. doi: 10.3390/jcm13123580 (PMC11205133; doi:10.3390/jcm13123580)
Supplement: Supplementary file 1 [file jcm-13-03580-s001.zip › jcm-3001286-Document S2.pdf]

Document S2. List of LMICs included in this review, as per the World Bank's 2023 GNI per capita classifications.

Low-Income Economies ($1,135 or less)	Lower-Middle-Income Economies ($1,136 – $4,465)	Upper-Middle-Income Economies ($4,466 – $13,845)
Afghanistan	Angola	Albania
Burkina Faso	Algeria	Argentina
Burundi	Bangladesh	Armenia
Central African Republic	Benin	Azerbaijan
Chad	Bhutan	Belarus
Congo, Dem. Rep	Bolivia	Belize
Eritrea	Cabo Verde	Bosnia and Herzegovina
Ethiopia	Cambodia	Botswana
Gambia, The	Cameroon	Brazil
Guinea-Bissau	Comoros	Bulgaria
Korea, Dem. People's Rep	Congo, Rep.	China
Liberia	Côte d'Ivoire	Colombia
Madagascar	Djibouti	Costa Rica
Malawi	Egypt, Arab Rep.	Cuba
Mali	Eswatini	Dominica
Mozambique	Ghana	Dominican Republic
Niger	Guinea	El Salvador
Rwanda	Haiti	Equatorial Guinea
Sierra Leone	Honduras	Ecuador
Somalia	Jordan	Fiji
South Sudan	India	Gabon
Sudan	Iran, Islamic Rep	Georgia
Syrian Arab Republic	Kenya	Grenada
Togo	Kiribati	Guatemala
Uganda	Kyrgyz Republic	Indonesia
Yemen, Rep.	Lao PDR	Iraq
	Lebanon	Jamaica
	Lesotho	Kazakhstan
	Mauritania	Kosovo
	Micronesia, Fed. Sts.	Libya
	Mongolia	Malaysia
	Morocco	Maldives
	Myanmar	Marshall Islands

	Nepal	Mauritius
	Nicaragua	Mexico
	Nigeria	Moldova
	Pakistan	Montenegro
	Papua New Guinea	Namibia
	Philippines	North Macedonia
	Samoa	Palau
	São Tomé and Principe	Paraguay
	Senegal	Peru
	Solomon Islands	Russian Federation
	Sri Lanka	Serbia
	Tanzania	South Africa
	Tajikistan	St. Lucia
	Timor-Leste	St. Vincent and the Grenadines
	Tunisia	Suriname
	Ukraine	Thailand
	Uzbekistan	Tonga
	Vanuatu	Türkiye
	Vietnam	Turkmenistan
	Zambia	Tuvalu
	Zimbabwe	West Bank and Gaza
